# Water-use efficiency of an old-growth forest in lower subtropical China

**DOI:** 10.1038/srep42761

**Published:** 2017-02-21

**Authors:** Xiaodong Liu, Xiuzhi Chen, Ronghua Li, Fengling Long, Lu Zhang, Qianmei Zhang, Jiyue Li

**Affiliations:** 1 Guangdong Key Laboratory for Innovative Development and Utilization of Forest Plant Germplasm, College of Forestry and Landscape Architecture, South China Agricultural University, Guangzhou 510642, China; 2 Key Laboratory of Vegetation Restoration and Management of Degraded Ecosystems, South China Botanical Garden, Chinese Academy of Sciences, Guangzhou 510650, China; 3 College of Natural Resources and Environment, South China Agricultural University, Guangzhou 510642, China

## Abstract

Carbon and water fluxes are key properties of ecosystem processes and functions. A better understanding of their temporal dynamics and coupling mechanism between these fluxes will help us improve ecosystem management for mitigation as well as adaption to future climatic change. From 2003 to 2009, carbon and water flux data were obtained by the eddy covariance method over an old-growth forest in the lower subtropical China. The 7 years of observational data indicated that the water-use efficiency (WUE) of the old-growth forest exhibited weak inter-annual variability. The mean annual WUE ranged from 1.70 to 1.98 g C kg^−1^ H_2_O. An analysis of the effects of environmental variables on the monthly gross primary productivity (GPP) and evapotranspiration (ET) indicated that solar radiation, air temperature, precipitation and vapor pressure deficit (VPD) produced similar effects on the monthly GPP and ET, which suggests that photosynthesis and ET were similarly driven by the climatic variables. At the monthly scale, the WUE decreased significantly as the precipitation and soil moisture content increased. However, a significant correlation was not detected between the WUE and the VPD at the monthly scale. Moisture conditions tend to be major drivers of the ecosystem WUE.

Monsoon evergreen broad-leaved forests (MEBFs) in southern China is the climax vegetation of the subtropics with a monsoon climate characteristics. In recent decades, however, droughts have become more frequent and severer because of the intensification of rainfall storms and the increasing number of annual rainless days caused by global climate change^1^, which have led to dramatic changes in the community composition and structure of MEBFs^2^. Carbon and water fluxes are key properties of ecosystem processes and functions^3^. Understanding the effects of carbon and water exchange on climatic and soil variables and their interactions is critically important for modeling and assessing the responses of vegetated systems to future climate change.

Carbon fluxes, including photosynthesis and respiration, are tightly coupled to water cycle in terrestrial ecosystems^4^,^5^. Water-use efficiency (WUE) is commonly defined as the ratio of gross primary productivity (GPP) to evapotranspiration (ET) for ecosystem-level studies^6^,^7^,^8^, and it is an important eco-physiological index that reflects the coupled relationship between ecosystem water and carbon cycles^9^. Previous studies have shown that WUE at the ecosystem level is typically controlled by climatic and soil variables, including precipitation, air temperature, vapor pressure deficit (VPD) and soil water content, because of their effects on energy partitioning and canopy conductance^8^,^9^, although the magnitude and direction of the response might be different or even opposite^10^. For example, Guo *et al*.^11^ found that high temperatures have a positive effect on crop WUE, whereas Li *et al*.^12^ reported that high temperatures have a negative effect on crop WUE. Krishnan *et al*.^13^ found drought enhanced growing-season WUE in an aspen forest, while Reichstein *et al*.^14^ proposed that drought has the opposite effect on WUE in a Mediterranean evergreen forest. Grünzweig *et al*.^15^ reported the same opposite effect on WUE in an Aleppo pine forest. These mixed results deeply reflected the complexity between carbon and water coupling at the ecosystem scale, which varies under different environments and vegetation characteristics.

Plants control the opening of their stomata to optimally satisfy the trade-off between the amount of carbon assimilated and the amount of water transpired^16^, and the constant diffusion coefficient of H_2_O is 1.6 times greater than that of the CO_2_ molecules^17^. At the ecosystem scale, however, seasonal and inter-annual climate variability and interactions between plant demands and soil supply for water and nutrients may differentially influence the GPP and ET and hence the WUE. Moreover, ecosystem ET involves water loss from both the canopy (transpiration) and the soil surface (evaporation) that inherently differ in response to changing water availability^18^. Therefore, understanding the variability of the GPP, ET and ecosystem WUE and the environmental factors that control these variables is valuable for evaluating the potential impacts of climate change on ecosystem carbon budgets and water resources.

Eddy covariance (EC) measurements represent a valuable approach for measuring carbon and water fluxes and examining WUE at the ecosystem level^19^,^20^. Based on EC technique, many studies have investigated the interactions between GPP, ET, and WUE in different forest ecosystems across China, including evergreen coniferous forests, deciduous broadleaf forests, evergreen broadleaf forests and the mixed forests^7^,^9^,^21^,^22^. However, limited by the number of flux towers in the subtropical area and the length of observation period^23^, previously related studies mainly focus on relatively short time-scales (e.g., diurnal, daily and seasonal), which inhibits our ability to accurately predict ecosystem carbon and water response to environmental change^24^. Long-term studies investigating the inter-annual variability in WUE and its environmental controls are therefore needed, especially for lower subtropical forests.

EC flux tower measurements have been performed in an old-growth subtropical forest in southern China since 2002 within the ChinaFLUX network. By looking into the importance of investigating carbon and water exchange and coupling in climatically sensitive subtropical forest ecosystem, we set-forth three objectives: (1) to analyze the seasonal and inter-annual variability in the GPP, ET and ecosystem WUE in old-growth subtropical forest for understanding the relationship between carbon gain and water consumption, (2) to estimate the effect of meteorology on variations in the monthly GPP and ET, and (3) to examine the environmental factors that control variations in the WUE.

## Results

### Environmental conditions from 2003 to 2009

**Figure 1:**
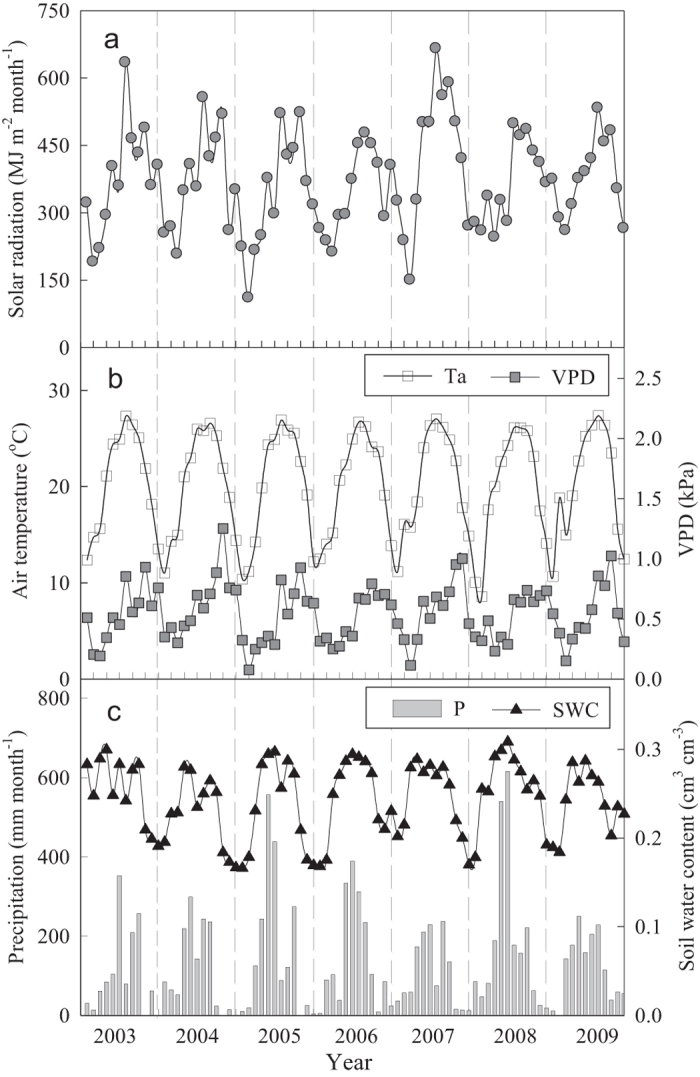
Seasonal variation in the (**a**) monthly solar radiation, (**b**) monthly mean air temperature (Ta) and monthly mean vapor pressure deficit (VPD) and the (**c**) monthly precipitation (P) and monthly mean soil water content (SWC) at a depth of 20 cm.

The seasonal patterns of the climatic variables and soil moisture are shown in Fig. 1. The monthly solar radiation was 371.2 MJ m^−2^ on average and ranged from 110.0 MJ m^−2^ to 665.0 MJ m^−2^ from 2003 to 2009. The solar radiation was highest in July, with a value of 535.1 ± 90.0 MJ m^−2^. The annual solar radiation varied from 4070.0 MJ m^−2^ (in 2005) to 5047.3 MJ m^−2^ (in 2007), and it was 2535.1 MJ m^−2^ during the wet season (April-September) on average and 1919.4 MJ m^−2^ during the dry season (October-March) on average. The mean annual air temperature was 20.2 ± 5.5 °C and ranged from 19.7 (in 2008) to 20.5 °C (in 2003). The vapor pressure deficit (VPD) varied between 0.25 kPa (during the wet month of March) and 0.92 kPa (during the dry month of October), with a mean of 0.54 kPa. At the annual scale, the mean VPD in 2004 and 2007 was greater than that in the other years because of the relatively lower precipitation and higher solar radiation. The pattern of solar radiation, air temperature and precipitation from 2003 to 2009 is representative of typical seasonal trends for this area, which presents warm wet summers and cool dry winters. Seasonal variations in soil moisture are closely tied to precipitation inputs, and the soil water content during the wet season (0.28 cm^3^ cm^−3^) was significantly higher than that in the dry season (0.21 cm^3^ cm^−3^) (*P* < 0.01).

### Seasonal and inter-annual variation in GPP, ET and WUE

**Figure 2:**
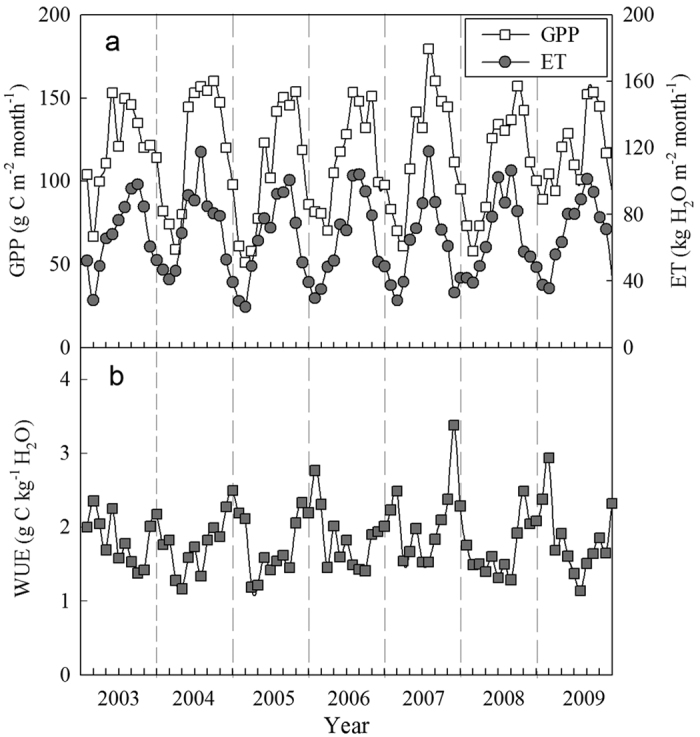
Seasonal patterns in the (**a**) gross primary productivity (GPP), evapotranspiration (ET) and (**b**) water-use efficiency (WUE) of an old-growth subtropical forest from 2003 to 2009.

The calculated GPP peaked in August from 2003 to 2009 and presented a mean value of 149.6 ± 7.3 g C m^−2^ month^−1^ (Fig. 2a). The monthly GPP maximum ranged from 152.9 to 179.5 g C m^−2^ month^−1^. On average, the GPP during the wet seasons was approximately 37.0% higher than that during the dry seasons and presented mean values of 798.8 ± 47.1 and 582.9 ± 39.3 g C m^−2^ for the wet seasons and dry seasons, respectively.

ET was the highest in July and presented a value of 98.7 ± 14.2 kg H_2_O m^−2^ month^−1^ during the 7-year monitoring period. The mean ET during the wet season was 505.3 ± 14.4 kg H_2_O m^−2^, which was approximately 73.6% higher than that during the dry season (291.0 ± 30.0 kg H_2_O m^−2^). At the inter-annual scale, the annual ET varied from 738.9 kg H_2_O m^−2^ year^−1^ (in 2007) to 835.7 kg H_2_O m^−2^ year^−1^ (in 2004).

The results of the regression analysis showed that the monthly GPP and ET from 2003 to 2009 were positively correlated (*y* = 1.096 *x* + 42.35, *R*^2^ = 0.67, *P* < 0.01). The strong correlation between GPP and ET demonstrated that the carbon and water cycles are coupled in the old-growth subtropical forest, which resulted in seasonal variations in the WUE (Fig. 2b). The annual WUE in the old-growth subtropical forest from 2003 to 2009 ranged from 1.70 g C kg^−1^ H_2_O to 1.98 g C kg^−1^ H_2_O. The annual average WUE was 1.83 g C kg^−1^ H_2_O and presented mean values of 1.60 ± 0.11 g C kg^−1^ H_2_O during the wet season and 2.03 ± 0.11 g C kg^−1^ H_2_O during the dry season (Table 1).

### Impact of climatic variables on GPP and ET

**Figure 3:**
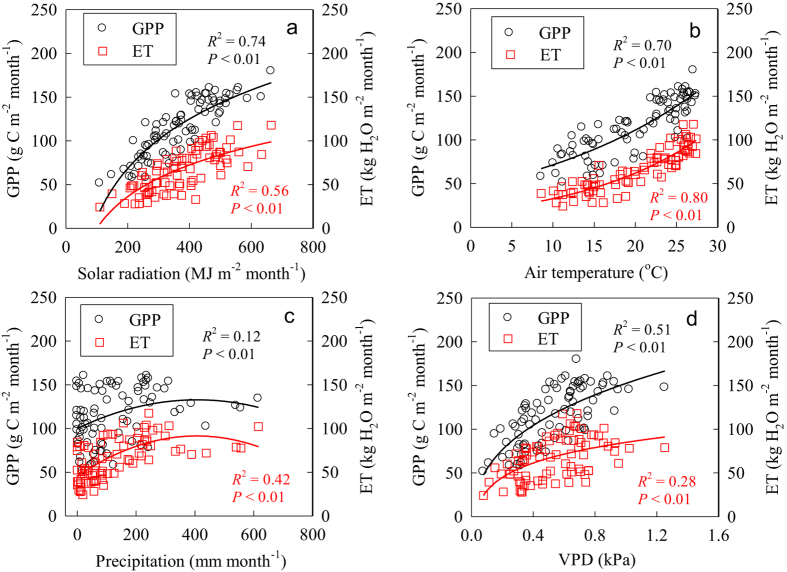
Relationship between the monthly gross primary productivity (GPP) and evapotranspiration (ET) and the (**a**) monthly solar radiation, (**b**) monthly mean air temperature, (**c**) monthly total precipitation and (**d**) monthly mean water vapor pressure deficit (VPD) in an old growth subtropical forest from 2003 to 2009.

**Figure 4:**
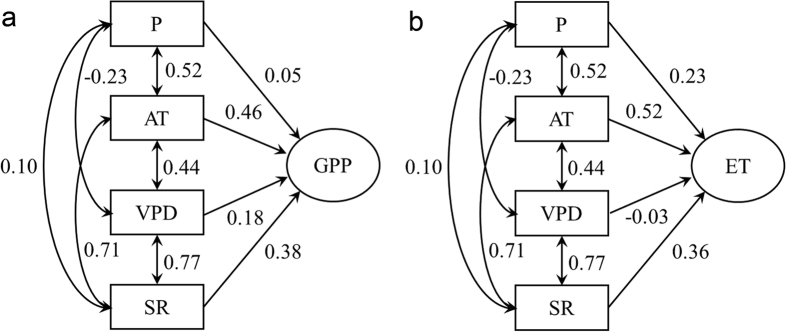
Path diagram illustrating the changing effects on climate variables related to the temporal patterns of monthly (**a**) gross primary productivity (GPP) and (**b**) evapotranspiration (ET) in an old growth subtropical forest from 2003 to 2009. Standardized correlation coefficients are labeled in the figure. P, AT, VPD and SR are the monthly precipitation, monthly mean air temperature, monthly mean water vapor pressure deficit and monthly solar radiation, respectively.

At the monthly scale, the total GPP and ET were strongly correlated with solar radiation, air temperature, precipitation and VPD, and they exhibited similar responses (Fig. 3), suggesting that photosynthesis and ET were driven by climatic variables some degree. As the main driver of photosynthesis and transpiration, solar radiation can explain 74% of the seasonal variation in GPP and 56% of the seasonal variation in ET. The monthly total GPP and ET both increased exponentially as the mean air temperature increased (*P* < 0.01). Air temperature explained 70% and 80% of the seasonal variation in GPP and ET, respectively. The relationships between GPP and ET and precipitation can be expressed by quadratic equations (*P* < 0.01), whereas the relationships between GPP and ET and VPD are best fit with logarithmic functions (*P* < 0.01). Besides, results from the path-analysis (Fig. 4) showed that the direct effects of air temperature and solar radiation were similar in shaping the temporal patterns of GPP and ET, which were higher than that of precipitation and VPD in magnitude.

### Impact of environmental factors on WUE

**Figure 5:**
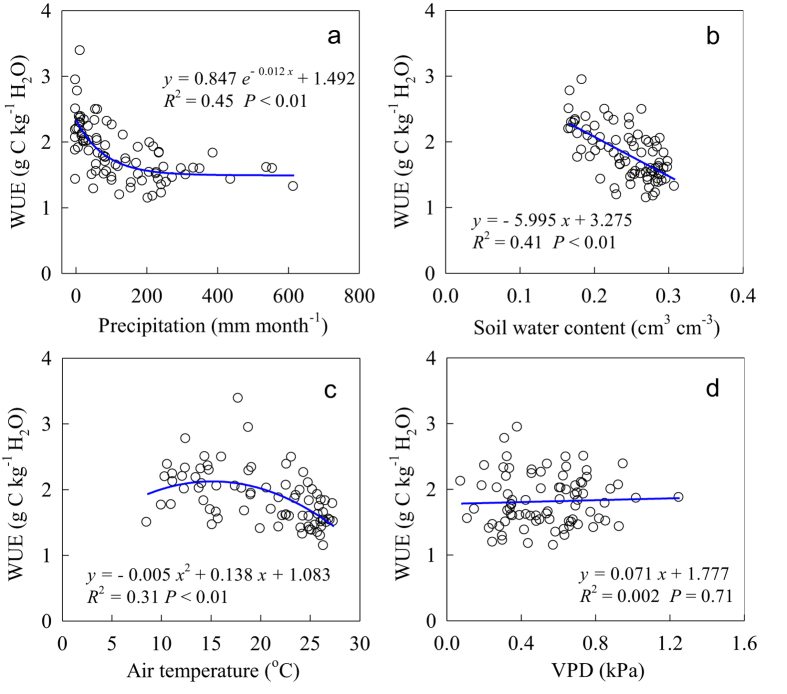
Relationship between the ecosystem water use efficiency (WUE) and the (**a**) monthly precipitation, (**b**) monthly mean soil water content at a depth of 20 cm, (**c**) monthly mean air temperature, and (**d**) monthly mean water vapor pressure deficit (VPD) from 2003 to 2009 in an old-growth subtropical forest.

**Figure 6:**
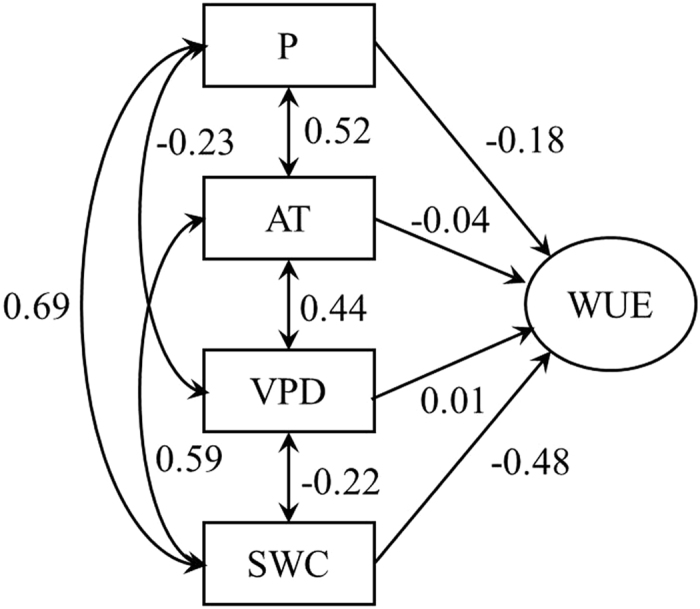
Path diagram illustrating the changing effects on climate variables related to the temporal pattern of ecosystem water use efficiency (WUE) in an old growth subtropical forest from 2003 to 2009. Standardized correlation coefficients are labeled in the figure. P, AT, VPD and SWC are the monthly precipitation, monthly mean air temperature, monthly mean water vapor pressure deficit and monthly mean soil water content at a depth of 20 cm, respectively.

During the 7-year experimental period, the WUE in the old-growth subtropical forest decreased exponentially as precipitation increased at the monthly scale (*P* < 0.01). The WUE showed a larger initial response through ET to wetting, whereas the GPP showed a comparatively weak response to wetting (Fig. 5a). At the monthly scale, the relationship between the ecosystem WUE and air temperature can be best fit by quadratic functions (*P* < 0.01) (Fig. 5b). Changes in the WUE were tightly coupled to changes in the moisture content of the upper (0–20 cm) soil profile. A strong negative linear relationship (*R*^2^ = 0.41, *P* < 0.01) was observed between the ecosystem WUE and soil moisture content in this subtropical environment (Fig. 5c). The relationship between the WUE and VPD was not significant (*P* = 0.71) (Fig. 5d). Results from the path-analysis (Fig. 6) showed that the temporal patterns of ecosystem WUE was largely affected by the direct effect of soil water content and precipitation, which were higher than that of air temperature and VPD in magnitude.

## Discussion

### Ecosystem carbon and water coupling

Carbon and water fluxes are key properties of ecosystem processes and functions. Their coupling processes are complicated over terrestrial ecosystems^3^,^24^. The eddy covariance method provides a convenient and efficient method of evaluating this coupling relationship between water and carbon cycles across different vegetation types at the ecosystem level worldwide^25^,^26^,^27^. During this 7-year experiment period, GPP was significantly correlated to ET at the monthly scale (*R*^2^ = 0.67, *P* < 0.01) and showed a strong linear relationship between C gain and water loss. Moreover, the monthly total GPP and ET exhibited similar responses to the climatic variables, especially solar radiation and air temperature.

WUE represents the trade-off between carbon gain and water consumption during the process of plant photosynthesis, and it exhibited relatively slight inter-annual variability in the old-growth subtropical forest. The annual average WUE from 2003 to 2009 was 1.83 g C kg^−1^ H_2_O, which was lower than the values reported by Yu *et al*.^7^ for a conifer plantation forest (2.53 g C kg^−1^ H_2_O) and a deciduous broadleaf forest (2.57 g C kg^−1^ H_2_O). The annual average WUE in our study was also lower than the values reported by Rodrigues *et al*.^28^ for a *Eucalypt* plantation and the values reported by Tang *et al*.^24^ for a mixed forest in Michigan (2.18 g C kg^−1^ H_2_O) (Table 2). The lower WUE in the old-growth subtropical forest was primarily attributed to the relatively mature stand (age > 100 years), which presented a lower GPP^20^ but higher ET because of the unique subtropical monsoon climate in southern China of abundant rainfall and consistently warm temperature^1^. Brümmer *et al*.^29^ reported that the WUE was in the range of 2.6–3.6 g C kg^−1^ H_2_O at most forest sites across an east–west continental-scale transect in Canada. Xiao *et al*.^22^ obtained WUE values between 0.87 and 3.59 g C kg^−1^ H_2_O from 22 sites across China. Essentially, WUE varies with the type of terrestrial ecosystem and differs strongly depending on the location, climatic factors, vegetation characteristics and disturbance regime^19^,^30^.

### GPP and ET in relation to climatic variables

Terrestrial ecosystem GPP is strongly affected by several abiotic and biotic factors, which can vary at different time scales^31^,^32^,^33^. At the monthly scale, the total GPP from 2003 to 2009 was significantly affected by the total solar radiation and precipitation, the mean air temperature and the VPD. The total monthly solar radiation could explain 74% of the seasonal variation in GPP; thus, solar radiation was expected to be one of the most important climatic variables governing GPP. Understandably, plant photosynthesis only occurs in environments with sufficient quantities of photosynthetically active radiation^34^. The annual GPP in the old-growth subtropical forests was quite steady and presented relatively limited inter-annual variation (1381.7 ± 65.0 g C m^−2^ year^−1^). This finding is consistent the results obtained by Yan *et al*.^35^ for a tropical seasonal rain forest (age > 180 years).

Similar to carbon fluxes, the monthly ET was also strongly correlated with the monthly mean temperature, solar radiation and total precipitation. Especially, Air temperature can explain 80% of the seasonal variation in ET. Both the monthly ET and GPP increased exponentially as the mean air temperature increased (*P* < 0.01). The exponential relationship with air temperature in this study was consistent with the results reported by Singh *et al*.^3^ for a subtropical pine (*Pinus roxburghii*) forest and Tong *et al*.^9^ for a warm-temperate mixed plantation. Here, the stronger ET and air temperature relationship than ET–VPD relation (Fig. 3d), clearly indicates that this ecosystem is more energy-limited than water-limited^3^,^36^.

### Response of ecosystem WUE to environmental factors

WUE is of considerable importance when investigating site-specific water cycles and the effect of drought on the water balance and C sequestration^10^,^13^,^37^. In this study, we found that the ecosystem WUE decreased with monthly precipitation, with strong decreases in WUE observed at precipitation levels of up to 200 mm and declining slowly thereafter. This finding is consistent with the results reported by Singh *et al*.^3^ for a subtropical pine (*Pinus roxburghii*) ecosystem and Yu *et al*.^7^ for a synthesis of three different ecosystems along a terrestrial transect in eastern China. Furthermore, enhanced WUE in dry years in the old-growth subtropical forest was also suggested. For example, during the dry year of 2007, the WUE was slightly higher than that during the years before the 2007 drought (i.e., 2003–2006). In 2007, the GPP in the old-growth subtropical forest was approximately 1433.8 g C m^−2^ year^−1^, whereas in the pre-drought years, the average was approximately 1376.2 g C m^−2^ year^−1^ (2003 to 2006). In addition, a greater amount of annual rainfall reduced the annual GPP because of a higher fraction of cloudy days^20^, whereas the annual ET decreased from 801.3 kg H_2_O m^−2^ year^−1^ to 738.9 kg H_2_O m^−2^ year^−1^ (Table 1).

Increases in temperature may increase or decrease the photosynthetic rate of plants and subsequently influence the accumulation of dry matter. However, temperature changes influence ET at the ecosystem level by affecting leaf stomatal conductance and soil evaporation^12^. Our study indicates that the ecosystem WUE exhibited a “saddle pattern” as the monthly mean air temperature increased. This result is inconsistent with the negative linear trend reported by Li *et al*.^12^ for a sparse vineyard in arid northwest China and the negative linear trend reported by Niu *et al*.^27^ for a temperate steppe. Zhou *et al*.^38^ suggested that plants may prioritize resistance to high temperature via opening the stomata unequally to improve the transpiration of leaves and dissipate heat at the cost of low WUE.

Soil moisture is expected to be the primary factor that controls the exchange of water and carbon between the land surface and atmosphere^39^. Our study also indicates that the WUE of the old-growth subtropical forest was negatively related to soil water content at the monthly scale, which is consistent with previous studies^9^,^40^. Many studies at the ecosystem level have also confirmed that under moderate drought, canopy conductance will decrease and ecosystem WUE will increase, whereas under extreme drought stress, WUE would then decrease because of the limited capacity for electron transport and carboxylation^14^,^41^,^42^.

A negative relationship between WUE and VPD has commonly been reported for ecosystem or leaf-level studies^19^,^43^,^44^. However, our study indicated that as the VPD increased in the wet and dry seasons, a significant response was not observed in the WUE. Increases in VPD influenced ET, especially transpiration, through a purely physical effect (a larger gradient of vapor pressure between the leaf air spaces and the atmosphere) and a biological effect (response of stomatal conductance to VPD)^42^. However, photosynthesis is mediated by changes in stomatal conductance. Thus, ET and photosynthesis were driven by VPD to similar degrees in the old-growth subtropical forest.

Our findings have important implications for understanding climatic change effects on carbon and water processes in subtropical ecosystems. With the projected decrease in soil moisture because of the intensification of rainfall storms and the increasing number of annual rainless days in southern China^1^, we expect that the WUE of the old-growth forest ecosystem will consequently increase at the ecosystem level. However, elevated temperature may increase or decrease ecosystem WUE at the monthly scale. As a consequence, changes in ecosystem WUE under climatic change will depend on the relative impact of concurrent changes in precipitation and temperature in this area.

## Conclusions

Here, we presented data on the seasonal variations in the GPP, ET and ecosystem WUE for an old-growth subtropical forest in southern China. In addition, the biophysical responses of these variables to climatic factors and soil moisture were also evaluated. The results from this study demonstrate that GPP and ET were similarly related to climatic variables (e.g., solar radiation, air temperature, precipitation and VPD), suggesting that variations in climatic factors controlled photosynthesis and ET to similar degrees at the monthly scale. Based on the 7-year experimental data, the WUE in the old-growth subtropical forest exhibited relatively slight inter-annual variations and presented a mean annual value of 1.83 ± 0.12 g C kg^−1^ H_2_O. The relatively low WUE was primarily attributed to the relatively mature stand that presents a lower GPP but higher ET in lower subtropical China. The ecosystem WUE was significantly correlated with precipitation, soil water content and air temperature but not VPD. This study will aid in predictions of the influence of global warming and drought stress on carbon and water fluxes in zonal forests of subtropical China.

## Methods

### Site description

The study was conducted in southern China at the Dinghushan Biosphere Reserve (DBR) (112°31′E and 23°10′N), which is approximately 90 km west of Guangzhou City, Guangdong Province. The reserve was established in 1950 to protect the natural monsoon broad-leaved evergreen forests in the lower subtropics and accredited as the first National Natural Reserve in China in 1956. The reserve has a total area of 1156 ha and experiences a typical subtropical humid monsoon climate and an average annual temperature of 20.9 °C. The highest and lowest extreme temperatures are 38.0 °C and −0.2 °C, respectively. The annual average precipitation is 1678 mm (1956–2009), and it has a distinct seasonal pattern, with approximately 80% of the precipitation falling during the wet season (April-September) and the remainder falling during the dry season (October-March)^1^. Soils are classified in the Ultisol group and the Udult subgroup according to the USDA soil classification system. The soil profile usually ranges from 50 to 80 cm in depth, with soil pH-values ranging from 4.5 to 6.0.

Because of the subtropical monsoon climate and the long history of protection in the DBR, this ecosystem contains well-protected old-growth forest (monsoon evergreen broadleaved forest) and various prophase successional forests. The monsoon evergreen broadleaved forest is greater than 400 years in stand age. Dominant species located in the central portion of the reserve include *Castanopsis chinensis, Canarium tramdenum, Schima superba, Cryptocarya chinensis, Cryptocarya concinna*, and *Machilus chinensis*. The height of the canopy is approximately 22 m, and the mean leaf area index (LAI) is 4.9 during the dry season and 5.6 during the wet season.

### Experimental measurements

Eddy covariance (EC) measurements of carbon and water fluxes and the associated environmental factors were collected from 2003 to 2009 at a study site in the forest stand with a fetch of greater than 5 km in every direction. The observation mast (cross-section 80 cm × 80 cm) was 38 m tall, the eddy covariance measurement height was 27 m, and the sensors used for the measurements included a CO_2_/H_2_O analyzer (Model LI-7500, Li-Cor Inc., NE, USA) and a three-dimensional ultrasonic anemometer (CSAT3, Campbell Scientific, Inc., Logan, UT, USA), which were used to measure fluctuations and averages of the wind velocity, temperature, CO_2_ and water vapor concentrations. The data were sampled at 10 Hz, averaged over 30 min, and directly recorded using the synchronous device for measurement (SDM) technique with a data logger (CR5000, Campbell Scientific, Inc.).

Along with the flux measurements, standard meteorological data were collected, including precipitation (52203; R.M. Young), solar radiation (CM11, CNR1; Kipp & Zonen), temperature, humidity, vapor pressure (HMP45C; Campbell and IRTS-P: Apogee), soil temperature (105-T and 107-L; Campbell) and soil moisture (CS616; Campbell). All of the routine meteorological data were recorded as 30-min mean values with the data loggers (3 CR10X and 1 CR23X; Campbell).

### Data processing and WUE calculation

To ensure the reliable processing of flux data, ChinaFLUX has developed a series of proven methodologies for carbon and water fluxes data quality assessment and quality control^23^. In this study, a three-dimensional rotation of the coordinates was applied to the wind components to remove the effect of sensor tilt or irregularity on airflow^45^. The Webb-Pearman-Leuning (WPL) correction was then used to eliminate the influence of air density fluctuations caused by the transfer of heat and water vapor^46^. We omitted abnormal data that were measured during periods of inadequate turbulence (u* < 0.2 m s^−1^). Data gaps were filled using nonlinear regression methods^47^. Additional details on the flux data quality assessment and quality control can be found in Yu *et al*.^23^. The percentage of missing or rejected data was 14% for this site.

GPP was calculated as follows:


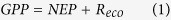


where NEP denotes the net ecosystem productivity (=−NEE, g C m^−2^ timescale^−1^), which was converted from the net ecosystem exchange measured by the eddy covariance system. *R*_*eco*_ was calculated as the sum of daytime ecosystem respiration and nighttime ecosystem respiration and estimated from empirical equations derived from soil temperature at a depth of 5 cm and water content in the top 10 cm of soil^7^.

ET was measured directly by EC technique. Daily ET was calculated as the sum of the H_2_O flux (kg H_2_O m^−2^ timescale^−1^).

Ecosystem WUE was calculated as follows:


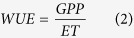


### Statistical analysis

Data analyses were carried out using the SAS (version 9.2, SAS Institute, Inc) software. The generalized linear model of regstats was used to conduct the regression analyses between GPP, ET and WUE and environmental variables and test the significance of the regressions, which were also conducted with nonlinear regression. By comparing the R^2^ and root mean squared error, we selected the better-fit functions that had a higher R^2^ and lower root mean squared error^48^. The path-analysis was conducted to evaluate the dependence of the temporal variations of GPP, ET and WUE on environmental factors.

## Additional Information

**How to cite this article:** Liu, X. *et al*. Water-use efficiency of an old-growth forest in lower subtropical China. *Sci. Rep.* **7**, 42761; doi: 10.1038/srep42761 (2017).

**Publisher's note:** Springer Nature remains neutral with regard to jurisdictional claims in published maps and institutional affiliations.

## Figures and Tables

**Table 1:** Gross primary productivity (GPP, g C m^−2^ timescale^−1^), evapotranspiration (ET, kg H_2_O m^−2^ timescale^−1^) and water use efficiency (WUE, g C kg^−1^ H_2_O) during the wet season (April-September) and dry season (October-March) in an old-growth subtropical forest.

Year	GPP (g C m^−2^ timescale^−1^)		ET (kg H_2_O m^−2^ timescale^−1^)		WUE (g C kg^−1^ H_2_O)	
	Wet season	Dry season	Wet season	Dry season	Wet season	Dry season
2003	815.4	626.3	487.8	326.9	1.70	2.00
2004	849.2	580.4	531.1	304.6	1.60	1.91
2005	740.8	528.2	499.4	265.8	1.47	2.01
2006	784.3	580.4	497.3	292.2	1.62	2.06
2007	868.5	565.3	498.4	240.5	1.77	2.18
2008	767.9	558.2	516.2	289.3	1.50	1.89
2009	765.4	641.8	506.9	318.0	1.53	2.13
Average	798.8	582.9	505.3	291.0	1.60	2.03
Coefficient of variation (%)	5.9	6.7	2.8	10.3	6.8	5.3

**Table 2:** Comparison of the WUE in this study with the WUE values for various forest types reported in the literature.

Forest types	Latitude, Longitude	MAT (°C)	MAP (mm)	WUE (g C kg^−1^H_2_O)	References
Boral aspen	53°42′N, 106°12′W	0.4	467	3.70	Krishnan *et al*.^13^
Douglas fir	49°54′N, 125°22′W	8.6	1451	5.40	Ponton *et al*.^19^
Maritime pine	44°42′N, 0°46′W	12.5	924	1.69	Berbigier *et al*.^26^
Ponderosa pine	44°30′N, 121°37’W	8.4	595	2.97	Law *et al*.^37^
Eucalypt plantation	38°38′N, 8°36′W	16.2	570	2.87	Rodrigues *et al*.^28^
Conifer plantation forest	26°44′N, 115°03′E	17.9	1485	2.53	Yu *et al*.^7^
Deciduous broadleaf forest	42°24′N, 128°05′E	3.6	695	2.57	Yu *et al*.^7^
Deciduous broadleaf forest	30°30′N, 120°30′E	15.7	1158	1.87	Wang *et al*.^15^
Evergreen broadleaf forest	28°36′N, 80°40′W	21.7	1294	2.35	Tang *et al*.^24^
Evergreen broadleaf forest	41°42′N, 12°22′E	16.0	778	3.13	Tang *et al*.^24^

MAT: mean annual air temperature; MAP: mean annual precipitation.
